# Position Accuracy Improvement by Implementing the DGNSS-CP Algorithm in Smartphones

**DOI:** 10.3390/s16060910

**Published:** 2016-06-18

**Authors:** Donghwan Yoon, Changdon Kee, Jiwon Seo, Byungwoon Park

**Affiliations:** 1 School of Aerospace Engineering, Sejong University, Seoul 05006, Korea; donghwan@sju.ac.kr; 2 Institute of Advanced Aerospace Technology, School of Mechanical and Aerospace Engineering, Seoul National University, Seoul 08826, Korea; kee@snu.ac.kr; 3 School of Integrated Technology and Yonsei Institute of Convergence Technology, Yonsei University, Incheon 21983, Korea; jiwon.seo@yonsei.ac.kr

**Keywords:** smartphone, android, location-based system, global navigation satellite system, differential GNSS

## Abstract

The position accuracy of Global Navigation Satellite System (GNSS) modules is one of the most significant factors in determining the feasibility of new location-based services for smartphones. Considering the structure of current smartphones, it is impossible to apply the ordinary range-domain Differential GNSS (DGNSS) method. Therefore, this paper describes and applies a DGNSS-correction projection method to a commercial smartphone. First, the local line-of-sight unit vector is calculated using the elevation and azimuth angle provided in the position-related output of Android’s LocationManager, and this is transformed to Earth-centered, Earth-fixed coordinates for use. To achieve position-domain correction for satellite systems other than GPS, such as GLONASS and BeiDou, the relevant line-of-sight unit vectors are used to construct an observation matrix suitable for multiple constellations. The results of static and dynamic tests show that the standalone GNSS accuracy is improved by about 30%–60%, thereby reducing the existing error of 3–4 m to just 1 m. The proposed algorithm enables the position error to be directly corrected via software, without the need to alter the hardware and infrastructure of the smartphone. This method of implementation and the subsequent improvement in performance are expected to be highly effective to portability and cost saving.

## 1. Introduction

The Global Navigation Satellite System (GNSS), which was initially limited to military or surveying fields, has gradually expanded into ordinary industrial fields such as navigation and time synchronization. As the price of GNSS chipsets has fallen below US$1 and smartphones have become increasingly popular, the number of GNSS applications, such as car navigation, geo-tagging, and location-based systems, is likely to rise dramatically. Various programs and services use positional information provided by the GNSS modules of smartphones. Indeed, 88% of American smartphone owners now use their handset as a map or navigation device in place of a car navigation kit [1], and the integration of visual reality/artificial reality (VR/AR) into real-world geolocation is expected to be realized in the near future [2]. As the positioning accuracy of GNSS chipsets improves, existing devices are likely to be replaced and location-based services will become further diversified. Automated vehicle identification, accident surveys, and emergency response require the location accuracy to be better than 4 m, and search and rescue and fire management are possible when a device is accurate to within 1–5 m [3]. Land surveys, such as for planning, topographic survey reconnaissance, geological surveys, and epidemiological mapping, require accuracy at the 1–3 m level and could therefore benefit from the use of smartphones [4]. Thus, there is strong interest in enhancing the achievable accuracy of smartphones, but the underlying positioning accuracy has remained unchanged over the past decade.

**Figure 1:**
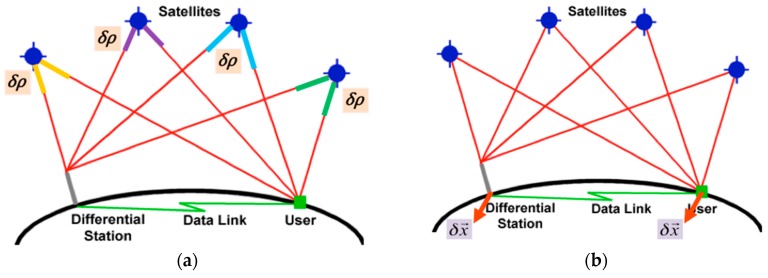
Range-domain DGNSS (**a**) and Position-domain DGNSS (**b**).

Smartphones are essentially personal computers with various sensors, including a GNSS module. Most smartphones can access the internet and run third-party applications. The first version of the Android smartphone, T-mobile G1, included a 528 MHz ARM 11 CPU and used the 3G or Global Service for Mobile communication (GSM) network. Smartphone-related technologies have expanded at a rapid pace: the latest device, the Samsung Galaxy S7, contains a quad-core 2.3 GHz CPU and receives data streams over Long Term Evolution (LTE) networks. The performance of GNSS chipsets has also improved. Assisted GPS (A-GPS) can obtain a faster location fix by acquiring almanac and ephemeris information via the cellular network. To provide more accurate and responsive location data to mobile users, even in the most challenging of environments such as urban canyons, recent smartphones have integrated the existing GPS-based location platform with the Russian GLONASS system since 2011 [5] and with China’s Beidou since 2013 [6]. Using this tri-band multi-constellation GNSS module, pedestrians and vehicles in urban areas can locate their positions for 95% of the day and those in harsh urban canyon (region in the red ellipse of the Figure 1) can find the locational information for 79% of the time. This is a considerable improvement over the figures of 56% and 20%, respectively, using GPS only [7].

The location chipset supporting the multi-GNSS positioning can improve the position accuracy and extend the position-available time. Table 1 presents static test results from DL-V3 (Novatel, Calgary, AB, Canada) receivers [8]. According to Seo’s results, integrating GLONASS into the GPS positioning system improves the root-mean-square (RMS) and mean value of the errors by approximately 18%, and the maximum error of 9.5 m has been reduced to 6.4 m. The RMS result for GPS stand-alone error has been improved to 0.42 m by applying a differential technique, and the performance of Differential GNSS (DGNSS) is better (by approximately 15%) than that of Differential GPS (DGPS). Thus, we conclude that DGNSS can provide more accurate and robust positional solutions than DGPS. Similarly, if an adequate DGNSS solution could be applied to smartphones, we would expect the position accuracy of the multi-GNSS chipset to be improved over that of the previous GPS-only chipset.

As described in the next section, there have been many attempts to improve the GNSS performance of smartphones to the level of DGNSS. However, smartphone and GNSS chipset vendors do not allow general users to feed the DGNSS correction to the positioning module or access the raw GNSS pseudorange. Thus, current applications remain purely conceptual approaches. In this paper, we consider the single-point positioning (SPP) algorithm of a GNSS chipset, and suggest a practical solution for improving the position accuracy.

The remainder of this paper is structured as follows: Section 2 discusses the motivation behind this work and lists the most important contributions. Section 3 describes the assumptions and verification process of the algorithm for the GNSS chipset considered in this study. We also introduce the proposed solution for implementing DGNSS suitable for the smartphone chipset. The results of static and dynamic tests to verify that our algorithm works well with the smartphone are presented in Section 4. We conclude this paper in Section 5 with a discussion and present some ideas for future work.

## 2. Previous Work and Contributions

### 2.1. Previous Work

The 95th percentile of horizontal/vertical accuracy in the GPS standard position service (SPS) is 3.4/4.7 m [9]. Similarly, the accuracy of the GPS modules embedded in smartphones is typically 3–5 m under good multi-path conditions; otherwise, it can be above 10 m. There have been several studies on improving the 5–10 m accuracy to the performance of DGNSS or RTK (Real Time Kinematics). These studies can be categorized into two groups: hardware add-on or modification methods and user-developed software.

A GPSWorld magazine article published in 2015 presented the results of an experiment in which a signal received through an antenna embedded in a smartphone was input to an external software-developed receiver. This report indicated that it is technically possible to achieve accuracy at the level of 10 cm [10]. To this end, however, it is impossible to implement or apply methods that allow smartphone manufacturers or users to improve the GPS accuracy of their devices directly without hardware modification. Moreover, the GPS modules in smartphones provide neither raw measurements (e.g., pseudorange measurements) nor an open port capable of receiving DGPS corrections. As current smartphones simply embed the GPS modules provided by the chip vender, the necessary functionalities are unlikely to be included until the vendors predict that a new market can be opened up by adding the function or the related module. In this respect, the approach of adding an independent DGNSS/RTK-enabled receiver seems to be more practical. Recent smartphones support the installation of user-developed applications, high-speed processing, and wireless communication to provide a new environment for the Network Transport of RTCM via Internet Protocol (NTRIP). Thus, a smartphone application that feeds DGNSS or even RTK corrections to a GNSS receiver connected to the smartphone via Bluetooth could be developed [11].

Although connecting an independent receiver to the smartphone would guarantee the positional accuracy, many studies have focused on developing suitable applications that use position information from the GPS modules of smartphones, which will improve portability and reduce costs. To determine the current location with similar precision to a more expensive DGPS solution, a correction is computed by subtracting the difference between the true and observed values at the known point. This is then applied to the coordinates of the features that are surveyed in that session [4,12].

### 2.2. Motivations and Contributions

DGNSS is a relative positioning technique that involves two receivers, namely a reference station (RS) and a user receiver. For real-time operations, the correction, which is generally broadcast via radio transmission or mobile communication [13], can be applied in two ways: “Position-domain DGNSS” or “Range-domain DGNSS”, as shown in Figure 1.

Range-domain DGNSS is more general and effective than Position-domain DGNSS, and corrections for the Range-domain DGNSS are provided free to all DGNSS users in Korea. One of the Korean DGNSS service providers, the National Maritime PNT Office, developed and distributed a service program running on smartphones to feed corrections to general GNSS receivers [14]. This cannot be used for the GPS module of smartphones, because Android applications can only provide the final position coordinates through LocationManager or GPS_Provider, and do not have the authority to access the GPS device [15]. Therefore, DGNSS techniques or the post-processing of SPP cannot be performed to correct or mitigate the error in the observable values, which translates to a positioning error. The only way to enhance the position accuracy of the smartphone is to shift the coordinates in the position-domain. Conceptually, Position-domain DGNSS is simple enough to mitigate the error in the smartphone position, because the correction is generated by differentiating the real-time GPS-derived position and the surveyed location of the RS. Although it seems possible to apply the block-shift method [16], this application can only be executed when the RS and the rover have exactly the same sets of visible satellites. When the two sets are different, the error is actually larger than for standalone GPS, and thus the block-shift method is impractical [17]. To overcome this problem, Lawrence considered both the RS and the user to be operating a smartphone and showed an overall improvement in accuracy over Ogundipe’s work; however, the subsequent errors were occasionally greater than 25 m [12]. The inverted DGPS method [18], in which the position of the user and satellite combination are sent to the server and the correction information is sent to the user, can be considered. However, this cannot handle multiple users, as the server would become overloaded.

The DGPS coordinate projection (DGPS-CP) method has recently been developed [19]. Under DGPS-CP, the rover selects the pseudorange correction (PRC) corresponding to the satellite combination used for rover positioning and projects it to the position area to correct its own position. This method is practical in smartphones, because the coordinate correction for visible satellites is generated at the user-side and the range-domain DGPS infrastructure (such as national DGPS (NDGPS) and the Satellite-Based Augmentation System (SBAS)) is used without modification. This technique is also convenient, as it uses the position standard format provided by smartphones, allowing users to apply the method within applications. In this paper, the DGPS-CP method is modified for smartphones, and the subsequent improvement in accuracy is verified through static and dynamic testing.

## 3. Approach and Implementation

### 3.1. Target Smartphone Specification

The target devices used in this study were a Galaxy S5 (Samsung Electronics, Suwon, Korea) and LG V10 (LG Electronics, Seoul, Korea), which were released in April 2014 and October 2015, respectively. The Galaxy S5 includes a Qualcomm MSM8974AC Snapdragon 801 Chipset running Android OS v4.4.2 (KitKat). The Snapdragon 801 processor obtains location information from IZat Gen8B [20] using a tri-band location platform that supports GPS, GLONASS, and BeiDou. The LG V10 has a Qualcomm Snapdragon 808 MSM8992 running Android 5.1.1 (Lollipop), and contains an IZat Gen8C [21]. This platform also supports GPS, GLONASS, and Beidou.

**Figure 2:**
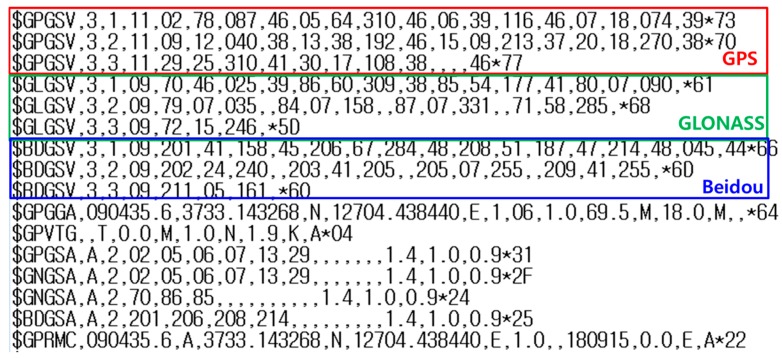
NMEA Position Output Example for a Samsung Galaxy S5.

Figure 2 shows the GNSS Satellite in View (GSV) message captured from National Marine Electronics Association (NMEA) data obtained by the Galaxy S5. The LG V10 captures similar logged messages. The GPGSV, GLGSV, and BDGSV data printed in the message represent the satellite IDs for GPS, GLONASS, and BeiDou, respectively. Thus, these three GNSS constellations are used to calculate the device’s position, and DGNSS corrections for GPS, GLONASS, and Beidou should be used together to improve the position accuracy.

The use of all three systems provides users with the benefits of observing more satellites in view and acquiring more reliable location information, but the imprecision of the time offset means that the position is not as precise as when the time systems are perfectly synchronized [22]. There are two approaches to mitigate this effect: use the time offset correction broadcast in the navigation messages, or introduce an additional unknown into the positioning solution [23]. Because the positioning algorithm is proprietary, and therefore not open to the public, we must guess which algorithm is used in the target device and check that this is correct in the field test experiment. 

### 3.2. Single Point Positioning Algorithm of the Smartphone

**Figure 3:**
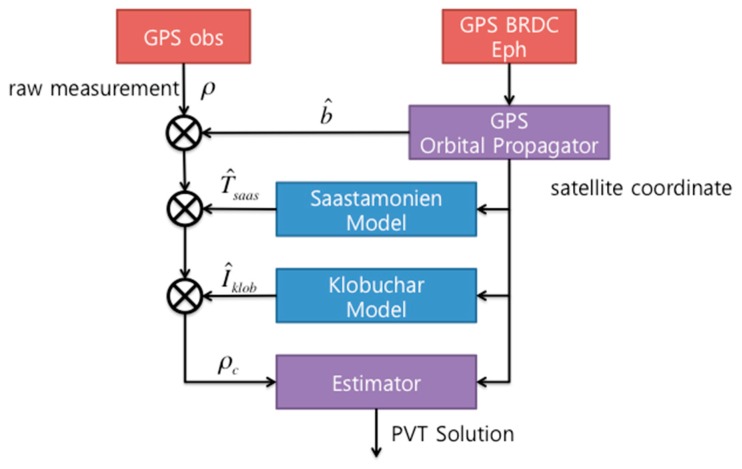
Typical algorithm for improving the GPS SPP Accuracy.

GPS SPP, also known as standalone or autonomous positioning, determines the user’s position via a single frequency receiver. SPP determines its own coordinates with respect to the center of the Earth by tracking four or more GPS satellites simultaneously. Its accuracy is generally poor, because errors in the broadcast (BRDC) ephemeris data and clock, as well as signal delay in the atmosphere, propagate into the signal. There have been many attempts to increase the accuracy of GPS SPP. The generally accepted technique is to remove the atmospheric delays using standard models [24,25,26,27]. Single frequency receivers typically use the Klobuchar Model according to the IS-GPS-200 method to calculate ionospheric delay [26], and the coefficients for modeling are included in the satellite navigation message [27]. Tropospheric effects are a function of the satellite elevation angle and the altitude of the receiver, and are dependent on the atmospheric pressure, temperature, and water vapor pressure [28]. As shown in Figure 3, the satellite clock offset (*b*) is obtained from the navigation message, and the atmospheric delays (${\hat{T}}_{saas}$ and ${\hat{I}}_{klob}$) are computed from standard models to obtain the error-reduced PVT (Position, Velocity, Timing) solution. 

The compensated pseudorange $\rho_{c}$ is calculated as shown in Equation (1), and this can be used in SPP in place of ρ to mitigate the positioning error.

(1) $$\rho_{c}=\rho -{\hat{T}}_{saas}-{\hat{I}}_{klob}$$

**Figure 4:**
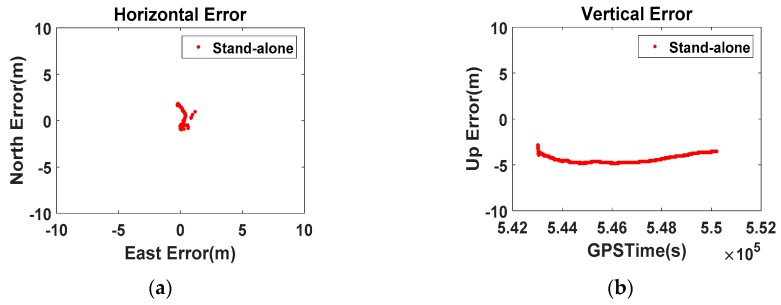
Horizontal and vertical error of the Galaxy S5 ((**a**) Horizontal; (**b**) Vertical).

Figure 4 shows the horizontal and vertical error variation logged from the Galaxy S5 smartphone at Sejong University RS. The RMS values (horizontal 1.4 m, vertical 4.4 m) are far smaller than those of the high-cost Novatel Flexpak receiver (horizontal 4.4 m, vertical 21.4 m). From these results, we inferred that the smartphone compensates for its pseudorange using the atmospheric model and Equation (1). The time offset between other GNSS constellations is a source of bias in multi-GNSS positioning, and can result in errors of up to 45 m after corrections using the parameters in the navigation message. The maximum error in the Galaxy S5 results is below 5.2 m, significantly less than 45 m. Therefore, we can assume that the module in this phone introduces an additional unknown parameter, namely the clock bias of each GNSS, into the solution. 

### 3.3. DGNSS-CP Algorithm for Android Smartphone

The DGNSS RS generates the Range-domain PRC ($\delta \vec{\rho }$) for each satellite as:

(2) $$\delta \rho^{i}={\hat{d}}_{RS}^{i}+{\hat{B}}_{RS}^{}-{\hat{b}}^{i}-\rho_{RS}^{i}$$

where:
$\delta \rho^{i}$:Range-domain PRC for i-th satellite${\hat{d}}_{RS}^{i}$:estimated distance from RS to i-th satellite${\hat{B}}_{RS}^{}$:estimated clock bias of RS${\hat{b}}^{i}$ :estimated clock bias of i-th satellite$\rho_{RS}^{i}$:pseudorange measurement for the i-th satellite received at RS

After receiving a bundle of PRCs from the RS, the rover selects a PRC combination ($\delta \vec{\rho }$) according to its visible satellite set. After applying the selected PRC combination to the observables ($\vec{\rho }$), the rover calculates the error-mitigated position by the least-squares method as:

(3) $$\left[\begin{array}{c} {\vec{x}}_{DGPS}\\ B \end{array}\right]={\left(H_{GPS}^{T}H_{GPS}\right)}^{-1}H_{GPS}^{T}\left[\begin{array}{c} \vdots \\ LOS_{ecef}^{i}\cdot {\vec{R}}^{i}-\left(\rho^{i}+\delta \rho^{i}\right)\\ \vdots \end{array}\right]$$

where:
${\vec{x}}_{DGPS}$:DGPS coordinates of the rover B:clock bias of the DGPS rover receiver$LOS_{ecef}^{i}$:line-of-sight unit vector of the i-th satellite in Earth-centered, Earth-fixed (ECEF) frame${\vec{R}}^{i}$:vector from the receiver to the i-th satellite$H_{GPS}$:observation matrix = $\left[\begin{array}{cc} LOS_{ecef}^{1} & -1\\ \vdots & \vdots \\ LOS_{ecef}^{n} & -1 \end{array}\right]$


Most DGPS correction messages for NDGPS (maritime applications), GBAS (Ground-Based Augmentation System) and SBAS (aviation) [29] are based on Range-domain DGPS. Typical DGPS devices provide raw observables or contain modules for reading and processing the correction. Android-based smartphones, however, do not provide the relevant authority for DGPS functions or access to the raw measurements. Thus, the Range-domain DGPS method must be modified to improve the position-domain accuracy of the smartphone. Under these considerations, DGPS-CP [19] offers a suitable DGPS solution for smartphones.

Similar to Range-domain DGPS, DGPS-CP selects a PRC combination ($\delta \vec{\rho }$) according to the satellite combination visible to the rover. To create a coordinate shift ($\delta \vec{x}$), DGPS-CP projects $\delta \vec{\rho }$ to the position area using the GPS observation matrix ($H_{GPS}$) obtained from Equation (3). This process can be written as:

(4) $$\delta \vec{x}={\left(H_{GPS}^{T}H_{GPS}\right)}^{-1}H_{GPS}^{T}\delta \vec{\rho }$$

To apply DGPS-CP to Android-based smartphones, we modified the existing DGPS-CP algorithm. First, we calculated the line-of-sight vector from the device to each visible satellite $LOS_{ecef}^{i}$ using the NMEA information of the smartphone, rather than the calculated satellite coordinates. We did not calculate the satellite position, because this would require a computationally intensive iteration process. Moreover, our approach avoids the need to obtain ephemeris parameters from the navigation message by accessing the Assisted GPS server, which is not generally open to the public. 

The GPGSV sentence in the NMEA data provides the azimuth angle (Az) and elevation angle (El). Unlike the existing DGPS-CP method, $LOS_{ecef}^{i}$ is obtained as shown in Equation (5), and the rotation matrix (R) is constructed using the latitude (φ) and longitude (λ) of the rover to convert $LOS_{local}^{i}$ to $LOS_{ecef}^{i}$, as shown in Equation (6):

(5) $$LOS_{local}^{i}={\left[\begin{array}{ccc} \sin\left(Az^{i}\right)\cos\left(El^{i}\right) & \cos\left(Az^{i}\right)\cos\left(El^{i}\right) & \sin\left(El^{i}\right) \end{array}\right]}^{T}$$

(6) $$LOS_{ecef}^{i}=R\left(\varphi ,\lambda \right)\cdot LOS_{local}^{i}$$

As shown in Equation (7), the *n* × 3 matrix E consists of $LOS_{ecef}^{i}$, where n is the number of satellites in each GNSS constellation:

(7) $$E={\left[\begin{array}{ccc} LOS_{ecef}^{1} & LOS_{ecef}^{2} & \begin{array}{cc} \cdots & LOS_{ecef}^{n} \end{array} \end{array}\right]}^{T}$$

Second, we considered multi-GNSS positioning for the DGPS-CP smartphone application. Both the Galaxy S5 and LG V10 use three constellations, as confirmed in Figure 4. In the previous section, we assumed that both devices obtain the time offset between the GNSS as one parameter of their navigation solution. Thus, to calculate the position-domain correction for the application, $H_{multiGNSS}$ was used instead of as the observation matrix $H_{GPS}$ in Equation (4):

(8) $$H_{multiGNSS}=\left[\begin{array}{ccc} E_{GPS} & -1 & \begin{array}{cc} 0 & 0 \end{array}\\ E_{GLONASS} & 0 & \begin{array}{cc} -1 & 0 \end{array}\\ E_{Beidou} & 0 & \begin{array}{cc} 0 & -1 \end{array} \end{array}\right]$$

Finally, we assumed that the GPS chipset in the smartphone uses the atmospheric model to improve its standalone position accuracy, as many commercial receivers do. Thus, it should be possible to restore the error mitigation in its pseudorange measurement. Using Equations (1) and (2), the compensated PRC ($\delta \rho_{c}^{i}$) was calculated according to Equation (9), and this quantity was substituted for $\delta \rho^{i}$ in Equation (4):

(9) $$\delta \rho_{c}^{i}=\delta \rho^{i}+{\hat{I}}_{klob}^{i}+{\hat{T}}_{saas}^{i}$$

## 4. Experiments and Results

### 4.1. Preliminary Test

**Figure 5:**
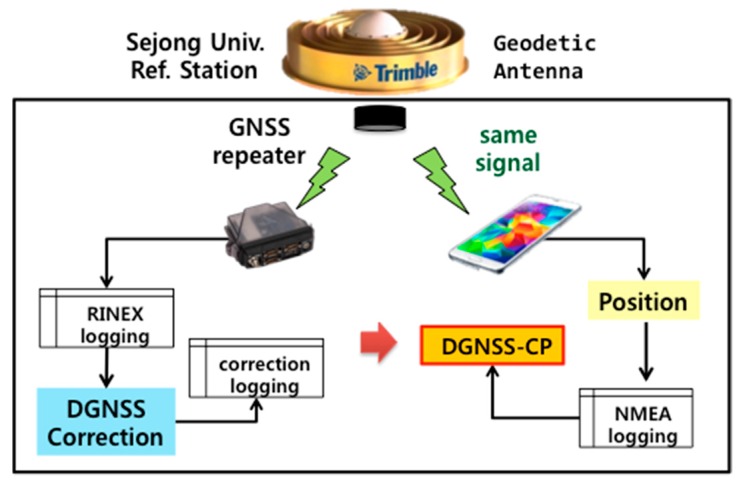
Zero-baseline static test setup.

To verify our assumption about the multi-GNSS positioning algorithm of the location chipset in the target smartphones, we conducted a preliminary test to shift the coordinates of the devices considering the conceptual position-domain DGNSS. In a zero baseline static test, the RS and a user device installed at the same location should observe the same satellites, and can theoretically cancel out all errors other than the thermal noise. Thus, this test is very useful for verifying the validity of the algorithm. Instead of a signal splitter, which is generally used for the zero-baseline static test, a GNSS repeater should be used, because smartphones cannot be connected to external antennas. We placed one Novatel FlexPak 6 receiver (as the RS) and one Samsung Galaxy S5 smartphone (as the DGPS user) under the GNSS signal repeater to construct the zero-baseline static test, as shown in Figure 5. The GNSS repeater was connected to the Trimble Zephyr Geodetic II antenna on the roof of the Chungmu building at Sejong University. Thus, both devices simultaneously received the same GNSS signal. This preliminary test was conducted from 16:00 to 18:00.

**Figure 6:**
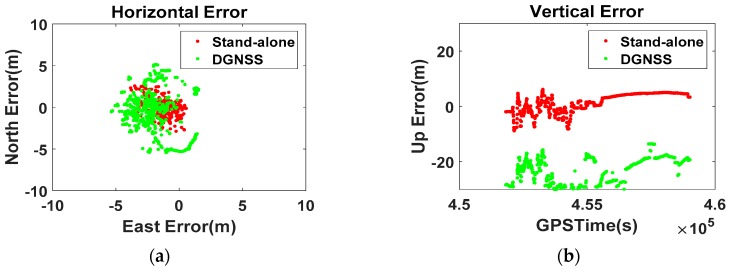
Zero-baseline Position-domain DGNSS results ((**a**) Horizontal; (**b**) Vertical).

Figure 6 shows the Position-domain DGNSS results given by the smartphone. The correction term was generated by differentiating the real-time stand-alone position of the RS from the precisely surveyed coordinates. The mask angle was assigned a value of 0°, and no atmospheric model was applied to the positioning options of the Flexpak receiver.

Even though the Position-domain DGNSS was applied to the smartphone, the RMS of the stand-alone error (horizontal 1.3 m, vertical 3.1 m) increased to 3.5 m and 24.7 m, and the maximum error reached 37 m. The RS real-time coordinates without an atmospheric model cannot improve the position accuracy of the smartphone, but rather degrades its performance, which gives reasonable proof of our assumption that the smartphone calculates its position after compensating for the pseudorange error using Equation (1).

Based on this assumption, we generated a new type of Position-domain DGNSS correction. Using the SETIONOTYPE and SETTROPOMODEL commands [30], we applied the Klobuchar Model and a troposphere model to the SPP. The new position-domain correction was generated by subtracting the model-applied real-time position from the surveyed coordinates. This is different from the generally known methods. 

**Figure 7:**
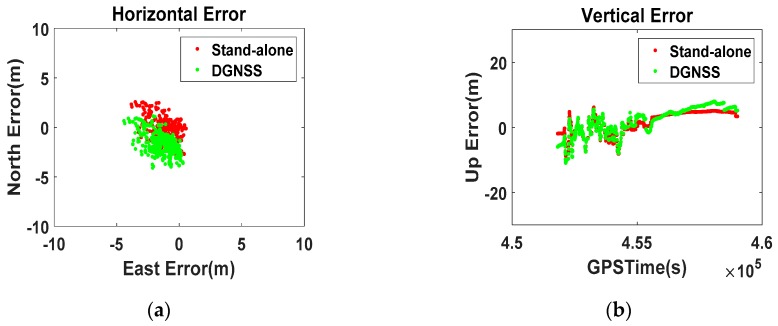
Zero-baseline Position-domain DGNSS results with the atmospheric model ((**a**) Horizontal; (**b**) Vertical).

The results in Figure 7 indicate that the errors were smaller than those in Figure 6, which means that compensated correction by the atmospheric model is more effective than the traditional method. Despite this advantage, the disagreement between the RS and the user regarding the visible satellites prevents further improvement in the stand-alone position result. This emphasizes the need to select the PRC set of visible satellites at the user-side, and that DGNSS-CP is the only method for correctly projecting the measurement-domain corrections onto the position-domain.

There were generally fewer satellites visible to the smartphone than to the RS, which confirmed our assumption that the smartphone uses a different satellite selection algorithm from that of the commercial receiver. For example, at the GPSTime of 458,095 s, GPS satellites G4 and Beidou satellite B2, B5, B6 were not used by the smartphone although they were observed at the RS. This disagreement increased the stand-alone error by 3.1 m, whereas the errors were bounded in the range 1–2 m when the two devices observed the measurements from the same set of satellites. 

### 4.2. Static Test

**Figure 8:**
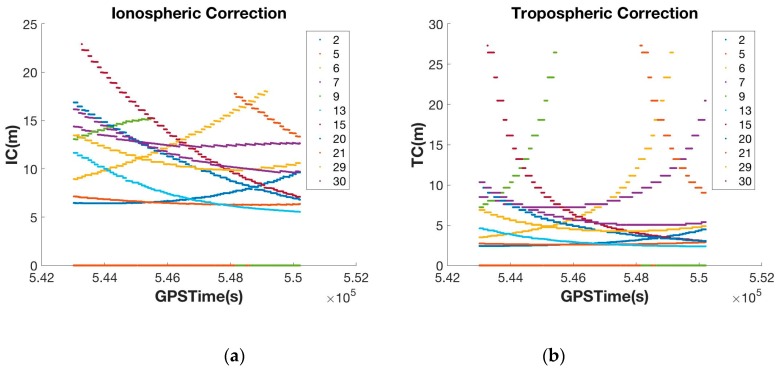
Ionospheric delay (**a**) and tropospheric delay (**b**) calculated by the atmospheric model.

To verify that the DGNSS-CP algorithm modified for the Android-based positioning works well in smartphones, another zero-baseline static test was conducted. The test setup was the same as in the preliminary test, and the V10 and Galaxy S5 were placed on the test-bed. We logged the raw data from the FlexPak 6 in Receiver INdependent EXchange (RINEX) format and the smartphone position output in NMEA format. By processing the measurements of the RS, we generated and logged the PRC in Radio Technical Commission for Maritime (RTCM) format. The Galaxy S5 experiment was conducted on 16 October 2015, and the V10 experiment was performed on 3 May 2016. Both experiments took place from 14:00 to 16:00, when the ionospheric variation is greatest. To calculate the empirical atmospheric correction in Equation (9), we used the Klobuchar and Saastamonien models. The parameters for the atmospheric model (see Table 2), four α values for the amplitude of ionospheric delay and four β values for the period of ionospheric delay [31], were taken from the RINEX navigation file and standard atmospheric model. Using the elevation angle information from the GSV data in the NMEA output, the ionospheric delay (${\hat{I}}_{klob}^{}$) and tropospheric delay (${\hat{T}}_{saas}^{}$) were calculated. The results are shown in Figure 8.

**Figure 9:**
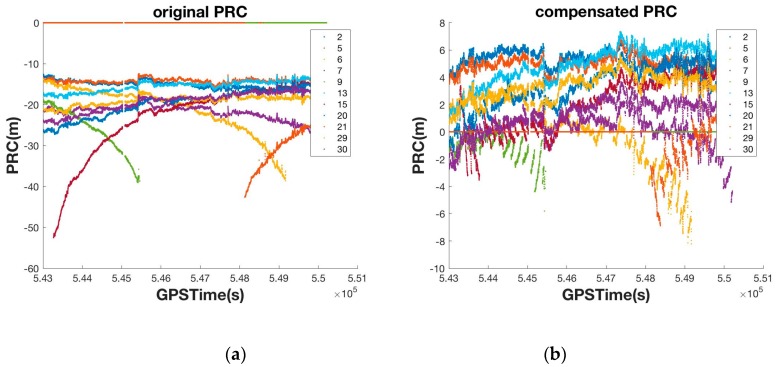
Original PRC in RTCM v.2 (**a**) and compensated PRC (**b**).

As shown in Figure 9, the original PRC generated by Equation (2) was over 50 m, but this was reduced to several meters using atmospheric compensation. This process was executed on the smartphone side, and the compensated PRC was then projected to the position-domain to create the coordinate correction ($\delta \vec{x}$).

**Figure 10:**
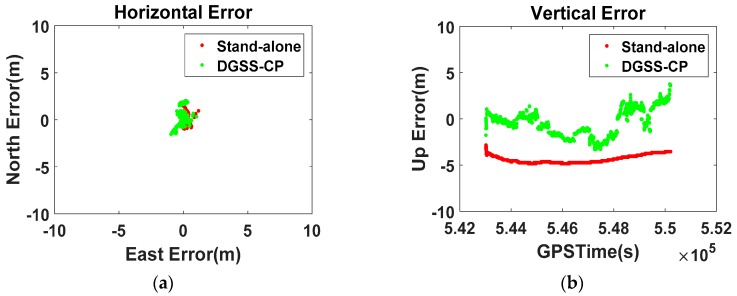
Zero-baseline static test results of the Galaxy S5 ((**a**) Horizontal error; (**b**) Vertical error).

The results of the zero-baseline static test for the S5 are shown in Figure 10. According to the horizontal position error on the left, the standalone result given by Android is about 1 m from the true position, whereas the modified DGNSS-CP (D/GPS + GLO + BDS) does not show such a noticeable bias. In the vertical error on the right, the state is displayed more distinctly. The standalone result shows an obvious bias of approximately 4.4 m, whereas, after applying the suggested algorithm, the vertical error fluctuates slightly around 0 m. As such, we can confirm that the proposed algorithm removes the error in the GPS position given by the smartphone. Additionally, it was confirmed that the noise in the standalone smartphone GPS result is far less than 1 m, which is the standard deviation of the ordinary GPS position error; this seems to be because a heavy filter was applied to make the performance of the chipset appear better than it actually is.

**Figure 11:**
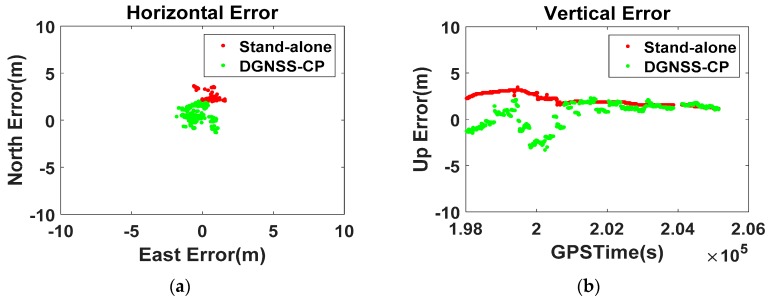
Zero-baseline static test results of the V10 ((**a**) Horizontal error; (**b**) Vertical error).

The V10 results in Figure 11 exhibit a similar tendency to those of the S5. The RMS of the horizontal and vertical error, 3.0 m and 2.3 m, respectively, has been reduced to 1.2 m and 1.3 m, as summarized in Table 3. The horizontal bias of 2.8 m has decreased to 0.7 m, and the maximum horizontal error of 3.7 m is now bounded at 2 m. At 199827 GPSTime, the RS generates the DGNSS corrections for all the visible satellites (GPS 4, 7, 8, 9, 11, 16, 21, 23, 26, 27, 30, GLONASS 10, 11, 20, 21, 22, 23, and Beidou 1, 2, 3, 4, 5, 7, 8, 10, 11, 12, 15), and based on the GSV messages V10 can select PRCs for the observed satellites (GPS 7, 8, 9, 11, 16, 21, 23, 26, 27, 30, GLONASS 10, 11, 21, 22, 23, and Beidou 1, 3, 4, 5, 10, 13) among the received PRCs. The smartphone then projects the set of range-domain PRCs to the position-domain using the modified DGNSS-CP algorithm to get a coordinate shift vector. As a result, the 3.3 m vertical error decreases to −0.2 m. 

### 4.3. Dynamic Test

**Figure 12:**
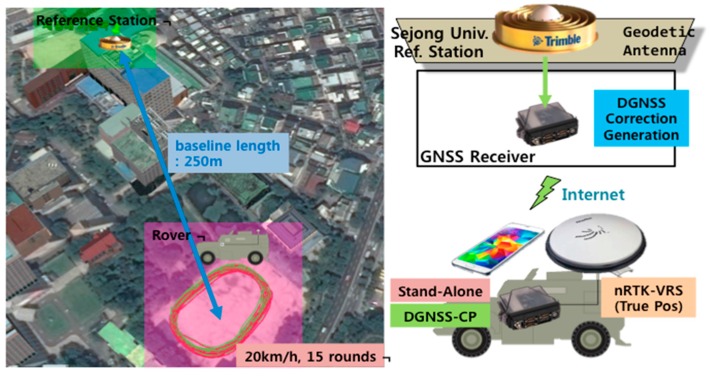
Dynamic Test Setup.

In a dynamic test, it is difficult to apply a heavy filter. Thus, a live performance comparison was conducted using a vehicle. An experiment using the S5 was conducted on 20 October 2015, from 20:20 to 20:30, and a test using the V10 was performed on 3 May 2016, from 01:35 to 02:00, when the ionospheric variation is relatively low. It was expected that any improvement in performance would be less pronounced than in the static test. The baseline from the RS was about 250 m, as shown in Figure 12, and the vehicle traveled around a playground at a velocity of approximately 20 km/h.

**Figure 13:**
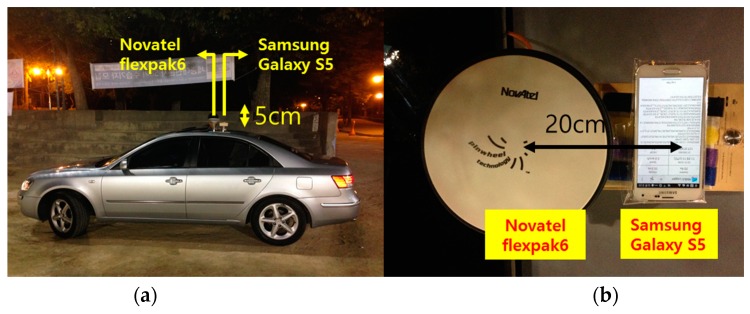
Configuration of the Automobile in the Dynamic Test ((**a**) Side view; (**b**) Top view).

A smartphone was placed on the roof of the vehicle, as shown in Figure 13, and the virtual reference station (VRS) service was applied to the Novatel FlexPak 6 receiver to obtain cm-level positioning; this was regarded as the true position (${\vec{X}}_{VRS})$ of the vehicle. The smartphone was placed 20 cm behind the antenna of the receiver. Thus, its location (${\vec{X}}_{smartphone})$ could be estimated using the velocity (${\vec{V}}_{VRS})$ of the vehicle and the horizontal difference (b) between the smartphone and the true position. This estimation process is described in Equation (10):

(10) $${\vec{X}}_{smartphone}={\vec{X}}_{VRS}-b\frac{{\vec{V}}_{VRS}}{\left|{\vec{V}}_{VRS}\right|}$$

**Figure 14:**
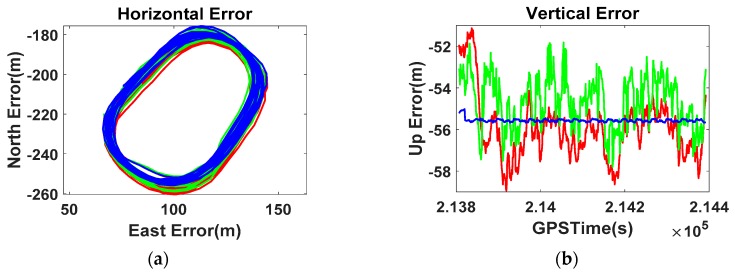
Trajectory of the dynamic test ((**a**) Horizontal trajectory; (**b**) Vertical trajectory).

According to the horizontal results shown in Figure 14, there was a considerable overlap between the VRS (blue) trajectory, which is assumed to be the true position, and the DGPS-CP result from the application (green). However, the standalone result was tilted slightly in a southwest direction. The vertical results confirm that the bias in the DGPS-CP position is smaller than that of the standalone device.

**Figure 15:**
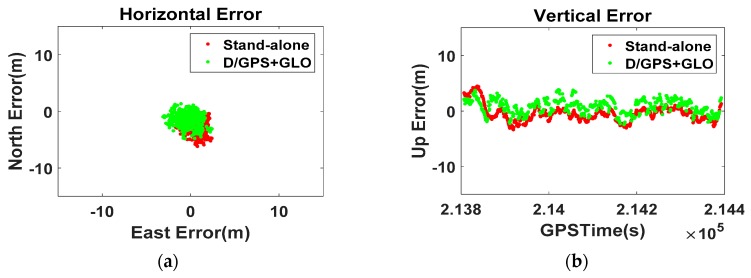
Dynamic test results of Galaxy S5 ((**a**) Horizontal error; (**b**) Vertical error).

The error components in Figure 14 are depicted in Figure 15. As previously identified, the standalone horizontal result exhibits an offset of approximately 2.8 m, whereas the bias of the corrected position has been reduced to 1.2 m. The resulting RMS of the horizontal error has decreased from 3.1 m to 1.9 m. The maximum error of 6.2 m has been reduced to 4.9 m after applying the DGNSS-CP algorithm, and the 2.8 m biased errors of the S5 have decreased to almost zero, only 1.38 m apart from the true position.

**Figure 16:**
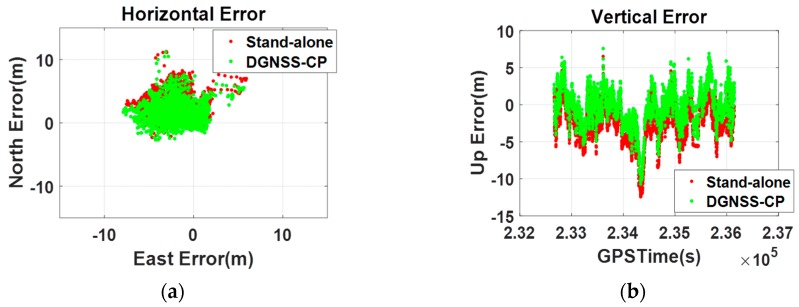
Dynamic Test Results of V10 ((**a**) Horizontal Error; (**b**) Vertical Error).

We can see a similar result in the V10 test results (Figure 16 and Table 4), where the horizontal error of 2.4 m and vertical error of 2.5 m have been reduced to less than 2.0 m. The 95th percentile of the horizontal positions is within a radius of 4 m. The mean values of the horizontal and vertical errors have been improved to 1.0 m and −0.2 m, respectively; therefore, the bias of the stand-alone position has been effectively mitigated. Compared to the 10 min test with the S5, the improvement in accuracy is relatively small, but the bias tends toward zero throughout the session. 

## 5. Conclusions

As smartphones have become more popular, GNSS has been used in various applications. The position accuracy of GNSS modules is one of the most significant factors in determining the feasibility of new location-based services for smartphones. The connectivity to the internet, capability of running third-party applications, and multi-GNSS module enable improved location performance in smartphones by implementing DGNSS functionality. Previous studies have attempted to shift the already-calculated coordinates using the RS real-time position, but they cannot guarantee position accuracies of 2–3 m in smartphones because of the disagreement between the satellite sets of the user and the RS. 

This paper proposed and implemented a DGNSS-correction projection method for commercial smartphones. First, the local line-of-sight unit vector was calculated using the elevation and azimuth angle provided in the position-related output of Android’s LocationManager, and this was transformed to Earth-centered, Earth-fixed coordinates for use by the correction method. In addition to GPS, data from the GLONASS and BeiDou satellite constellations were used for positioning. Thus, to achieve position-domain correction, the line-of-sight vector was transformed to an observation matrix suitable for multiple constellations. We identified the likely structure of the algorithm in the GNSS module of the smartphone, and demonstrated that this assumption was correct via several preliminary tests. This was an essential step in modifying the DGNSS-CP algorithm for use on the target smartphones.

The results of static and dynamic tests confirmed that the standalone Android GPS accuracy could be improved by 30%–60% using the proposed approach, and showed that the maximum error could also be reduced. In addition, the proposed algorithm does not require the infrastructure of DGPS correction to be modified, and the rover corrects its positional error based on the standard NMEA position output format, which can be acquired directly from the smartphone. Thus, the proposed method is very simple to implement. Therefore, if used to create simple programs, the proposed algorithm could significantly improve the position accuracy of current smartphones using software alone.

## Figures and Tables

**Table 1:** Analysis results of position accuracy for GPS, multi-GNSS, and differential methods.

	GPS(L1)-only		GLONASS-only		Multi-GNSS	
	SPP	Differential GPS	SPP	Differential GPS	SPP	Differential GNSS
RMS (m)	2.84	0.42	2.98	0.64	2.37	0.41
Mean (m)	2.39	0.36	2.58	0.44	1.99	0.30

**Table 2:** Parameters for the Klobuchar and Saastamonien Models.

**Ionospheric Parameters**	**α0**	**α1**	**α2**	**α3**
	$0.2049\times 10^{-7}\;$	$-0.7451\times 10^{-8}$	$-0.1192\times 10^{-6}\;$	$0.5960\times 10^{-7}$
	**β0**	**β1**	**β2**	**β3**
	$0.1249\times 10^{6}$	$-0.3277\times 10^{5}$	$-0.2621\times 10^{6}$	$0.1311\times 10^{6}\;$
**Tropospheric Parameters**	**Pressure (mb)**	**Temperature (°K)**	**Humidity**	
	1013.25	291.25	50%	

**Table 3:** Statistics of Static Test Results.

Statistical Results			Max	Mean	STD	RMS	95%
Galaxy S5	Stand-alone	Horizontal	1.84	1.19	0.65	1.35	1.83
		Vertical	4.83	−4.36	0.42	4.36	4.83
	DGNSS-CP	Horizontal	2.04	0.41	0.69	0.80	1.85
		Vertical	3.76	−0.42	1.45	1.27	2.70
LG V10	Stand-alone	Horizontal	3.69	2.83	0.88	2.96	3.78
		Vertical	3.45	2.30	0.60	2.28	3.17
	DGNSS-CP	Horizontal	2.13	0.73	0.93	1.19	2.08
		Vertical	3.3	0.20	1.38	1.25	2.44

**Table 4:** Statistics of Dynamic Test Results.

Statistical Results			Max	Mean	STD	RMS	95%
Galaxy S5	Stand-alone	Horizontal	6.24	2.76	1.41	3.10	5.26
		Vertical	4.48	−0.44	1.48	1.17	3.08
	DGNSS-CP	Horizontal	4.87	1.34	1.53	2.04	4.02
		Vertical	3.85	0.79	1.28	1.25	2.72
LG V10	Stand-alone	Horizontal	11.66	1.56	1.78	2.36	4.15
		Vertical	12.43	−1.83	2.44	2.53	6.38
	DGNSS-CP	Horizontal	10.61	0.96	1.83	1.98	3.96
		Vertical	10.7	−0.22	2.42	1.94	5.71
